# Use of antimicrobials and other medical products in an ethnic minority context of South-Central Vietnam: A qualitative study of vulnerability

**DOI:** 10.1371/journal.pgph.0002982

**Published:** 2024-04-09

**Authors:** Maya Ronse, Thuan Thi Nguyen, Xa Xuan Nguyen, Brecht Ingelbeen, Mira Leonie Schneiders, Duong Thanh Tran, Joan Muela Ribera, Charlotte Gryseels, Koen Peeters Grietens

**Affiliations:** 1 Department of Public Health, Institute of Tropical Medicine, Antwerp, Belgium; 2 Department of Malaria Epidemiology, National Institute of Malariology, Parasitology, and Entomology, Hanoi, Vietnam; 3 Department of Anthropology, Philosophy and Social Work, Rovira i Virgili, Tarragona, Spain; University of Oslo Faculty of Medicine: Universitetet i Oslo Det medisinske fakultet, NORWAY

## Abstract

Despite the global threat of antimicrobial resistance (AMR), evidence on the use and quality of medicines at community level is limited, particularly in impoverished, rural areas where prevalence of (bacterial) infections is high. To better understand the processes that drive vulnerability to AMR’ effects, this study aimed to assess social factors underpinning access to–and use of–medical products and healthcare, among people from the Raglai ethnic minority in Ninh Thuan Province, Vietnam. We conducted ethnographic research in eight villages in 2018–2019, using interviewing and participant observation methods for data collection. Different types of informants (including community members and healthcare providers) were selected using purposive sampling strategies and analysis was retroductive. Our findings show that, despite the existence of a government-funded health insurance scheme, Raglai people’s flexible therapeutic itineraries did not systematically start with formal healthcare. Different types of care (private/informal, public, shamanic) were combined in parallel or in alternation, determined by distance to the provider, cost, workload, perceived diagnostic capacity, perceived severity and aetiology of the illness, and trust in the provider. Available medicines were often tablets dispensed in plastic bags containing labelled tablets, unlabelled tablets (in bulk) or tablets ground to powder. Treatment was often considered effective when it relieved symptoms, which led to abandonment of the treatment course. When symptoms did not speedily abate, the illness aetiology would be reinterpreted, and “stronger” medicines would be sought. The precarious socio-economic status of some Raglai drove them in cycles of severe poverty when additional unforeseen factors such as illness, animal disease or loss of crops arose, hampering access to (in)formal healthcare providers and/or appropriate diagnosis and treatment. We conclude that Raglai communities are structurally unable to buffer themselves against the threat and consequences of AMR. Despite this vulnerability, they are among the least targeted by efforts to optimize antibiotic use, which are concentrated in secondary and tertiary healthcare facilities targeted at urban populations.

## Introduction

Antimicrobial resistance (AMR) is a public health threat of global importance. Exposure to antimicrobial (AM) substances contributes to the emergence and spread of AMR microorganisms (bacteria, viruses, parasites and fungi) [1]. The dynamic interaction between people (social actors such as patients and healthcare providers), health systems (access to healthcare and to AM treatment, infection prevention and control measures, policy and political context), and the environment (AM exposure in livestock, food, plants, water, soil and other household products) affects exposure to antimicrobials and therefore the biological emergence and spread of AMR. In addition to human AM use, use in animals, and exposure through agriculture also contribute to increasing rates of AMR [2–5].

In terms of antibiotic resistance (ABR) specifically, absence of hygiene and sanitation measures in resource-limited settings result in higher incidence of bacterial infections [6–10] and consequently in higher consumption of antibiotics. In resource-constrained settings, access to hospitals is often limited and antibiotics are frequently used at community-level. However, while antibiotic resistant blood-stream infections may be largely community-acquired, most reports and interventions to address ABR in these settings are hospital-based [11–14].

Inappropriate use of antimicrobials at community level can accelerate the emergence and spread of AMR, by increasing pathogen and commensal bacterias’ exposure to antimicrobials, out-selecting resistant bacteria, which are then more frequently transmitted [1, 3, 15–20]. Inappropriate use of antimicrobials is not limited to misuse or overuse [21], but also underdosing, related for instance to poor treatment adherence, poor storage of antimicrobials, poor quality antimicrobials, non-prescription, and certain treatment preferences [22, 23].

The use of medicines has been shown to be related to socio-cultural factors. These factors range from patients’ perceptions of healthcare providers and of the efficacy and quality of medicine, perceptions of illness severity and recovery, the choice for public or private providers, as well as an understanding of side effects and fears thereof. Additionally, broader contextual factors such as accessibility and quality of healthcare [24–26], (partially) influenced by the political and historical context, are other factors to consider. In addition, some broader socio-cultural factors also affect the context in which healthcare is sought and provided: actions and perceptions of governments and healthcare providers, healthcare sector regulations (e.g. over-the-counter dispensing of antibiotics), gaps between health policy and implementation, and between intended and actual service delivery, availability of different medicine dispensers in the public-private sector mix and medical pluralism practices, financial or geographical barriers to appropriate diagnosis and treatment, waiting times at providers, quality of medical training, and availability of medical personnel [25, 27–29]. Several of these factors also affect trust in the health system, the emergence of rumours regarding public health campaigns, and more general conceptions on health and illness [25, 27–29]. In addition, factors affecting access, such as financial constraints to pay for antimicrobials, or public stock outs, have also been described as barriers to appropriate antimicrobial use, resulting in an inverse association between income and AMR [27, 30, 31].

South-East Asia represents an important setting in terms of antimicrobial drug resistance due to a unique convergence of sociocultural, political-economic, ecological and biological factors. The region faces high levels of bacterial resistance to antibiotics [32–36], largely attributable to higher incidence of community-acquired infections [8–10] and has repeatedly been the epicentre of the emergence of antimalarials parasite resistance [33, 37–39].

In Vietnam particularly, increasing ABR has been documented in human and animal health [40–44]. A recent study in a rural community found that 80.6% of households had members carrying colistin-resistant E. coli [45]. Despite government regulations, antibiotics are generally dispensed without medical prescription [46]. Recent studies in Vietnam estimated the share of antibiotics dispensed without prescription between 55.2% and 91% [24, 47]. For mild illness, healthcare is reported to be mainly (72.5%) sought at drug stores, of which the majority are non-licensed, whereas for severe illness, care is sought at public facilities (74.1%) [24]. Vietnam has developed a national AMR action plan, including an antibiotic stewardship programme, but has not yet reported results to WHO’s Global Antimicrobial Resistance Surveillance System (GLASS) [48].

AMR is clearly shaped by the interaction between biological and human social processes [49, 50]. Our qualitative study therefore aimed to explore the social processes that drive vulnerability to the effects of AMR generally and to the suboptimal use of medical products specifically. As exposure to a range of infectious diseases as well as reduced access to appropriate healthcare often cluster in remote and sometimes marginalized ethnic minority populations in South-East Asia, our study focused on Raglai people’s access to healthcare and use of medical products. This study is part of a longstanding collaboration on health-related research in the area since 2004.

Although the initial objective was to assess perceptions and use of antibiotics and ABR, we found that the concepts of antibiotics and ABR as defined in a biomedical framework were not part of the language or reference framework of the study population. We therefore moved the focus of the research to the use of medical products and experiences of treatment failure in general, while keeping an analytical lens on AMR. This particular study aimed to assess social factors underpinning access to–and use of–medical products and healthcare, among people from the Raglai ethnic minority in Ninh Thuan Province, Vietnam.

## Materials and methods

### Study design

Our study consisted of a qualitative emergent-theory design based on ethnographic fieldwork [51].

### Study site and population

This study was carried out in eight villages of Bac Ai and Ninh Son districts, situated in the hilly and forested part of the Ninh Thuan province (South-Central Vietnam). In 2022, Bac Ai counted a population of approximately 31,000 inhabitants, predominantly of Raglai ethnicity, in addition to Cham and Kinh ethnicities, the latter being the Vietnamese majority population [52]. According to 2022 local statistics, the Ninh Son district had a population of over 61,000 inhabitants, predominantly of Kinh ethnicity in addition to various other ethnic groups including Cham, Raglai, Co Ho, and Nung [52]. Ninh Son is approximately 28 km from Bac Ai district.

Our interviews and observations were in majority with people who identified as being from Raglai ethnicity and refered to themselves as (the) “Raglai”. The Raglai are a diverse group of people, and when we will mention “(the) Raglai” in this work, we only refer to the study population we interviewed. Our Raglai informants took a certain pride in saying they were Raglai, and always asked us from where we came. Therefore, in referring to these informants and their communities in that way, we are trying to respect their identity.

People of Raglai ethnicity are categorized by the government as an ethnic minority group. Despite the government’s socio-economic interventions in the past 20 years, people of Raglai ethnicity are among the poorest groups in Vietnam [53]. Raglai people engage in small-scale subsistence slash-and-burn agriculture in the forests on the mountains, occasionally combined with livestock, hunting, and logging [54, 55]. In the last two decades, Raglai were relocated from ‘old villages’ in their traditional forested territory to ‘new villages’ constructed by the government as part of its policy for resettlement and socio-economic development of ethnic minority groups [56]. Public healthcare and schools are made available in ‘new villages’. Despite the government’s interventions and the gradual integration with the market economy, more than half of the district’s population–estimated at 58,6%–lives under the national poverty line [57].

Health studies found that people of Raglai ethnicity had a higher risk of malaria infection and malnutrition rates among children under 5 years of age in comparison to those who were of Kinh ethnicity [54, 58–60].

District hospital data from 2016–2018 reported an increasing trend of dispensed antibiotics at public health facilities [61]. Although there are no granular AMR data from Bac Ai district, a small hospital-based study at the provincial reference hospital for our study setting reported a high prevalence of ABR amongst bloodstream infections in 2017 (e.g. 100% resistance to ampicillin, 92.7% to trimethoprim/sulfamethoxazole, 72.7% to ciprofloxacin, and 72.0% to ceftriaxone in 144 E. coli bloodstream infections, preserving only carbapenems to treat these infections) [62]. Malaria parasite resistance was found in some provinces in south-central Vietnam including Ninh Thuan [63] and delayed parasite clearance has been spreading [64–66].

### Positionality

The research team was composed of two field researchers (TTN–female, MR–female). The Vietnamese researcher (TTN) was from Kinh ethnicity and had already been conducting fieldwork among predominantly Raglai communities for several months, acquiring familiarity with informants of Raglai ethnicity, gatekeepers in the field, the Raglai culture, and the Raglai accent in Vietnamese. Prior to the study, TTN conducted four periods of fieldwork in the study sites, between July 2016 and April 2018, as part of her PhD research on malaria transmission among Raglai population. Each fieldwork was between four and six weeks. Being from Kinh ethnicity, TTNs ethnographic approach was a requirement to gain trust and allow proper data collection among this ethnic minority. With the exception of a few people who only spoke Raglai, TTN could communicate directly with most informants in Vietnamese. The second Belgian researcher (MR) faced language barriers, needing translation, and initial increased socio-cultural distance. The prolonged stays in the communities, in combination with MR’s precedence from a “neutral” country–not involved in the Vietnamese war–and not part of inter-cultural socio-political tensions at national level, helped to gradually build trust and lead to informants opening-up about certain topics in a different way. Members of the larger study team (XXN and KPG) have been doing research embedded in this study area and population since 2004.

### Data collection

Two researchers conducted ethnographic fieldwork between April-August 2018 (MR and TTN) and May-July 2019 (TTN). Collected data consisted of semi-structured in-depth interviews (IDI), (focus) group discussions, and participant observation including informal conversations. The field researchers participated in everyday activities including observations at communal health centres and private practices as well as residing with community members. This allowed to build rapport and trust, and gain insights into living conditions, socio-cultural practices, people’s health-seeking behaviours, as well as their perceptions, use, and/or prescription of medicines. When IDIs and group discussions were recorded, this was done using a dictaphone. Sometimes only notes could be taken. For informal conversations, notes were written down as soon as possible after the conversation. All data were transcribed in either English or Vietnamese by TTN and MR with the assistance of a local field assistant.

### Sampling

We selected informants following theoretical and snowball sampling strategies. Both communities and participants were gradually selected within the study area on the basis of emerging information. Informants included community members (both from “old” and “new” villages); human health service providers (public and private sector, as well as informal providers); animal health service providers (public, private sector, and informal); representatives of local government (local authorities, forest protection guards etc.); and traditional power structures (shaman, respected “wise” persons). We aimed for maximum variation in occupational profiles (farmers, doctors, informal providers, grocery shop owners); ethnicities (Raglai, Cham, and Kinh); gender (female, male); and age groups (older people, adults, and children). Further details on the categories of informants are provided in S1 Table. Data collection resulted in 43 informal conversations in combination with participant observation, 71 IDIs, and 10 group discussions.

### Analysis

Analysis followed the principles of retroductive analysis [67]. We analysed data intermittently during, between, and after data collection phases. We carried out thematic content analysis and analysed transcribed data with the help of NVivo (11) software (QSR International Pty Ltd. Cardigan UK), based on themes pre-identified from the initial research questions in addition to new themes that emerged during intermittent analysis [68]. Data was coded and analysed by two authors (TTN and MR). A theoretical framework on vulnerability underpinned this work (cf. discussion).

### Ethical approvals

Ethical approval was granted by the Institutional Review Board of the Institute of Tropical Medicine (ITM), Antwerp (Belgium)–Ref 1234/18 –in addition to the Ministry of Health and the National Institute of Malariology, Entomology, and Parasitology (NIMPE), Hanoi (Vietnam)–IRB decisions 1648/QD-VSR and 3731, QD-BYT. We obtained oral consent from all informants. Participants had the opportunity to ask questions, voice concerns, or withdraw from participation at any time during the research. Specific attention was given by the researchers to detecting culturally specific “polite/indirect” signs of refusal to answer questions and/or to participation, in which cases researchers stopped the data collection. As an example, some individuals responded "I do not speak Vietnamese" as a means of declining to participate in the study, even though they had basic knowledge of the language. In other situations, people expressed interest in hearing our introduction, but politely declined when invited to participate in the study.

### Inclusivity in global research

Additional information regarding the ethical, cultural, and scientific considerations specific to inclusivity in global research is included in the S1 Checklist.

## Results

The findings presented hereunder are categorised in four main types of factors influencing either directly or indirectly access to–and use of–medical products and healthcare: factors influencing (1) patients’ access to healthcare; (2) patients’ choice of providers; (3) patients’ choice and use of medical products; and (4) factors driving diagnosis and prescribing practices of providers.

### Factors influencing patients’ access to healthcare

#### Subsistence strategies and financial resources

In our study setting, farming land assigned to Raglai people in the new villages was limited in size and prone to droughts. The economic pressure, escalating government regulations and control over forest resources (i.e. ban on creating new fields, extraction of timber products, and hunting in the protected areas) has led to changes in subsistence and mobility strategies of many informants, resulting in a combination of slash-and-burn agriculture in their old villages, rice farming in the new villages, and seasonal forest and plantation work. Key informants reported being constantly short of cash for essential needs and experienced seasonal food shortage as a result of severe droughts over the past few years. Forest extraction activities (vegetation, flowers, honey etc.) provided some income in between harvests, but also led to tensions between local (Raglai) community members and forest management authorities. To meet the demands of a changed lifestyle in the new villages, many informants took loans from the government and private lenders. Some people entered cycles of severe poverty when additional unforeseen factors such as illness, animal disease or loss of crops arose, hampering access to (in)formal healthcare providers and/or appropriate diagnosis and treatment.

#### Marginalization

Raglai were often sceptical and/or scared of people from the dominant ethnic group. Despite being proud of the traditions originating from Raglai culture and cosmology, including spiritual shamanism and the advantages of a matrilineal kinship system, Raglai informants often showed signs of low self-esteem when comparing themselves to other ethnic groups or foreigners. When being asked about their culture, Raglai informants often repeated the stereotypes developed by mainstream society, i.e. being “backward”, maintaining a forest-based lifestyle, practicing “backward” traditions such as shamanism, being physically “ugly” (i.e. having a darker skin tone compared to the ideal standards of fair skin tone in dominant Kinh ethnicity), wearing dirty working outfits, speaking with an unusual accent in Vietnamese (i.e. Kinh), being poor, and having low education.

These degrading stereotypes also play a role in the healthcare encounter. Health information materials available to Raglai people at health centers portrayed Kinh “ideals”, implying fairer skin tone, a small family size and signs of belonging to the “industrial” world (e.g. having an industrial job). References to local culture and people were sometimes used in promotion materials, such as representing people in traditional outfits, with forest-going/nomadic lifestyles, living in bamboo stilt-houses. However, these can be ambiguously interpreted as depicting stereotypes of ethnic minorities in Vietnam.

#### Accessibility of health information

Most of Raglai people could hold basic conversations in Kinh language but few could read or write. At health facilities, all printed health education materials were in Kinh. With the majority of health staff also being of Kinh ethnicity, communication between health staff and patients was mostly in Kinh, which many Raglai only partly comprehended. Communication was further complicated by the inter-ethnic hierarchy and the pressure on health professionals to shorten time spent on each patient in order to control waiting times. It was observed and shared in interviews that both public and private prescribers often did not provide clear information on diagnosis, aetiology, and instructions on treatment, especially when receiving mixed-medicine bags containing different types of tablets with different posologies.

#### Incongruent conceptualizations of pathogens and medicines

Biomedical causations of diseases and associated explanations provided by health staff often were difficult to translate into a locally adapted reference framework, with the absence of existing similar terms or concepts in Raglai language. As an example, in Raglai language, specific terms for antibiotics and bacteria, viruses, or parasites were non-existent and commonly referred to as ‘medicine’ *(jrãu)* and ‘worms’ (*ana hula*) respectively. In terms of symptoms for Raglai, fever *(sot)* and headache *(dau dau)* were considered interchangeable terms as they related to similar symptoms. It was suggested by a Raglai nurse that only Raglai with more medical literacy would be able to make out the difference. From a biomedical point of view, this added a level of complexity in explaining differences between these biomedical concepts and causations in diagnosis, and general health communication for Raglai.

### Factors driving patients’ choice of providers

Even though more than 90% of the population in the studied villages was estimated to have access to a basic health insurance provided by the government to populations living in poverty and remote areas [69], public health facilities were not always the first choice of care. Therapeutic itineraries for one health problem commonly combined several types of care, either in parallel or in alternation, which was influenced by a complex interplay of factors.

In this study setting, the range of healthcare options was relatively wide, offering many therapeutic options to communities, ranging from primary and secondary biomedical healthcare–including public and private commune health centres (CHC); hospitals; home practices by nurses, doctors, pharmacy technicians; sole dispensing of medical products by private pharmacies, grocery shops, and mobile sellers–to shamanic healing. See Table 1 for an overview of the available therapeutic options and the perceived comparative advantages in our study context.

**Table 1:** Therapeutic options and comparative (dis)advantages as perceived by informants in the study setting.

Different primary health care providers & dispensers	Perceived advantages	Perceived limitations
**Public Commune Health Centre**	• Free of charge (if holder of health insurance card) • Short geographic distance from most “new” villages • Chance of getting information and a diagnosis after assessing symptoms (sometimes) • If complication, level-up referral possible (covered transport for referral sometimes not possible) • (Sometimes incentives if part of a study)	• Limited range of drugs available (incl. injections/transfusions) • Slow effect of some drugs (products manufactured in Vietnam) • Long waiting lines and complex administrative procedures • Chance of lower cultural proximity with healthcare workers
**Public District Hospital**	• Perceived high efficacy • Free of charge medical care and hospital bed (if holder of health insurance card) • Free transport to district/provincial hospital if further referral needed • Wide range of medicines available (incl. transfusions and injections) • Chance of getting information and a diagnosis (sometimes) • Option of running tests	• Often longer distance • Long waiting lines and complex administrative procedures • Chance of lower cultural proximity with healthcare workers
**Private hospital**	• Perceived high efficacy • Perceived wider range of drugs and technologies • Reduced waiting times • Friendly environment and communication with patients • Attentive to patient’s needs	• High cost • Often longer distance • Profit making, not always for the best interest of patients
**Private (home) practices ^a^** **(by nurses, doctors, pharmacy technicians etc.)**	• Greater variety of medicines available than at health centres often (esp. transfusions) • Availability of “strong” and “fast”-working imported (foreign) medical products • Possibility to ask for a technical intervention only (e.g. come with own transfusion bag to receive the transfusion) • Partial regimens can be purchased based on financial means of patient • Possibility to pay later • Less waiting lines and no administrative procedures which saves time • Often closer geographically • Dedicated care, psychological support, cultural proximity	• Can be expensive • Dependent on the availability and “rules” of the practitioner in question
**Private pharmacies ^a^** **(by pharmacist assistants usually)**	• Greater variety of medicines available than at HC often • Availability of “strong” and “fast”-working imported (foreign) medical products • Partial regimens can be purchased based on financial means of patient • Allows for self-medication • No waiting lines and quick service • Perceived high efficiency of drugs • Possibility to pay later	• No diagnosis • Can be expensive • Can be located quite far from “new” villages
**Grocery shops ^b^**	• Fast, closest to where people live geographically • Selling a combination of drugs, vitamins, and other supplements (with similar or same labelling as drugs sold at pharmacies) pre-divided in small plastic bag per dosage • Partial regimens can be purchased based on financial means of patient • Allows for self-medication • No waiting lines and quick service • Possibility to pay later	• Limited choice of drugs • No diagnosis • No professional training • (less guarantee of quality e.g. expired drugs)
**Mobile sellers ^b^** (regularly passing by in villages on motorbikes)	• Fast, highly convenient for patients as no travel needed • Also sell other daily essentials • (In one village: sellers were from a trusted related ethnicity) • Partial regimens can be purchased based on financial means of patient • Allows for self-medication • Possibility to pay later	• Limited choice of drugs • No diagnosis • Lack of control over when mobile sellers pass by
**Shamanic healing**	• Only option to treat spiritual root causes of disease • Finds the cause of / person responsible for the problem (spiritual aetiology / sorcery) • Can contribute to restoring one’s wellbeing (the body, mind, and spirit) • Possibility to pay later • Dedicated care and psychological support • Part of Raglai identity and tradition	• Expensive (costs of animals for rituals) • Perceived by outsiders as a “backward” tradition

^a^ These providers/dispensers did not necessarily have a training for this as would be required in the public/official sector.

^b^ These dispensers did not have a training for dispensing medical products as would be required in the public/official sector.

### Time and work

In subsistence farming, which determined most Raglai’s life rhythm, time meant money and resources. As such, seeking care became a priority only when the symptoms compromised the ability to work. In this case, striving for time efficiency appeared to weigh most heavily in the decision where and with which provider to seek healthcare. Consequently, waiting lines in public health centres were perceived as an important impediment. In addition, while in theory there should always be staff on duty, in some health centres staff occupied post-office hours for emergencies only, and not for minor healthcare problems. For some community members, however, the perception of what was urgent or not was different from staff’s perceptions, leading to occasional disagreements that impacted on the relationship of trust between provider and patient. These experiences would then influence subsequent health-seeking itineraries.

### Distance and geographical accessibility

The time required to travel to a health facility was particularly important in remote and old villages where it takes a considerable amount of time to get out of the forest, down the mountain, on bad quality roads, to a pharmacy or a public health centre. Home practices, grocery shops, and mobile sellers provided a comparative advantage in this regard. Poorest families had to mainly travel by foot, while better off families had motorbikes. The motorbikes were often owned and used by men, which sometimes made it difficult for women to access care quickly in case of emergencies.

### Cost

To evaluate the affordability of a provider the following needed to be taken into account: (i) direct non-medical costs such as the financial costs of transport, (ii) direct medical costs of healthcare and medicines, as well as (iii) indirect costs such as the loss of income and food due to not being able to work; as well as the missed chores and (temporary) abandonment of relatives (children, older people, disabled) in need of care at home. All Raglai communities were entitled to free basic health insurance provided by the government due to their designation as ‘poor’ and remote residence, covering selective medical services in the public sector [70]. To obtain a health insurance card, people needed to officially register for a new card annually, except for children under six years of age who were exempt from the renewal requirement). Given the Raglai’s poverty, families that for various reasons did not obtain a health insurance card or did not go to the public health sector needed to use a high proportion of their total income to purchase medicines and health services. This sometimes led to the need to sell domestic animals, to borrow money from relatives or to take an individual loan from a grocery shop (often owned by someone from Kinh ethnicity) as a financial coping mechanism, which would subsequently become a major burden on the household. In such a context, the option of buying partial drug regimens, according to the means of the patient, or having the option of paying at a later time, were considered important advantages for health providers to attract clientele.

### Perceived diagnostic capacities of the provider

The possibility for receiving a diagnosis, an explanation for the cause of the disease and a description of the problem itself, was a valued advantage of biomedical healthcare providers, both in health centres and in private practices. Nevertheless, the speed of recovery was often deemed to be more important and therefore previous experiences relating to speed of service and recovery prognosis often outweighed the benefits of receiving a concrete diagnosis. People therefore often sought out those drug dispensers that were perceived to be more accessible and were known to have more effective and fast-acting drugs, regardless of their diagnostic capacities.

### Provider-patient relationship

Being welcoming, taking time to listen to the patient and giving clear information in a respectful way and comprehensible language (preferably Raglai), showing cultural proximity or sensitivity (more likely to be achieved by Raglai staff) were elements that were seen to foster trust from the patients and contribute to a good reputation of the healthcare provider. These perceptions were often circulated and fed back within the community, leading to a reputation of a provider which would either attract more patients in the future, or to the contrary, lead to avoidance by patients in case of negative experiences. Anecdotes were shared of patients being addressed by staff in disrespectful ways, such as being asked to sit further away during a consultation because of their unpleasant smell. References were also made to unequal inter-ethnic relations as healthcare workers were often from a different ethnicity than the Raglai patients in our study.

“*…In general the instructions [given by the district health centre] are easy for patients to understand*. *I am Raglai and they can understand what I say… Patients find your people [Kinh] annoying*. *My people rarely raise our tone when we speak to the patient, we always handle the conversations with them gently, we explain every little detail to them. Your people [Kinh] often give short explanations to the patients [which makes them] think they were being scolded.” (IDI, healthcare worker at a public health centre, 16/05/2018)*

### Perceived aetiology of the illness symptoms

In Raglai cosmology, humans are intrinsically connected to the environment they are born in, to the land that hosts living beings as well as their ancestors. All these connected elements are believed to have spirits. These spirits, including the own spirit of a person, can be offended in case of antisocial behaviour or disrespect towards essential traditional values, unnecessary destruction of natural resources, and trespassing of sacred spaces. Such behaviours can trigger ill health. “Bad” shamans were believed to be able to afflict illness as well. Both biomedical and spiritual aetiologies allowed for biomedical (“western”) treatment and/or spiritual shamanic healing (involving rituals, prayers, offerings/sacrifices) as appropriate courses of action as both could be intertwined. Herbal medicine is also part of traditional care, though it was mentioned that a lot of this knowledge was being lost among the younger generations. Biological diseases could be associated with “worms” in the body (imagery stemming from biomedical healthcare personnel ranging from viewable to invisible organisms in the body causing illness). Additionally, sudden ecological changes (e.g. in the weather and climate) in combination with intensive working conditions were also perceived to place a heavy burden on the body, leading to sickness or other disruptions in a natural balance such as changes in diets etc. Sudden changes in weather could refer to sudden rain, wind, and cold or on the contrary, to very high temperatures and sun exposure. However, this was also associated to perceptions of climate change, showing increasingly unpredictable changes in weather and seasons which was said to have a significant impact on agriculture practices and working conditions in the fields. Spiritual illness could be associated with conflicts between spirits and people (e.g. offenses to the ancestors) or between people (e.g. jealousy). The longer biomedical treatments remained ineffective in relieving symptoms, the higher the probability that people perceived either the medicines not to be appropriate in relation to the severity or the “strength” of the disease; or interpreted the illness to be caused by something which “Western medicine” could not solve. This was the case for complicated, relapsing, and chronic diseases, where aetiological associations with spirits and/or sorcery often became the final diagnosis, dedicating all efforts towards spiritual healing.

### Factors influencing patients’ choice and use of medical products

#### Administration route

**Fig 1:**
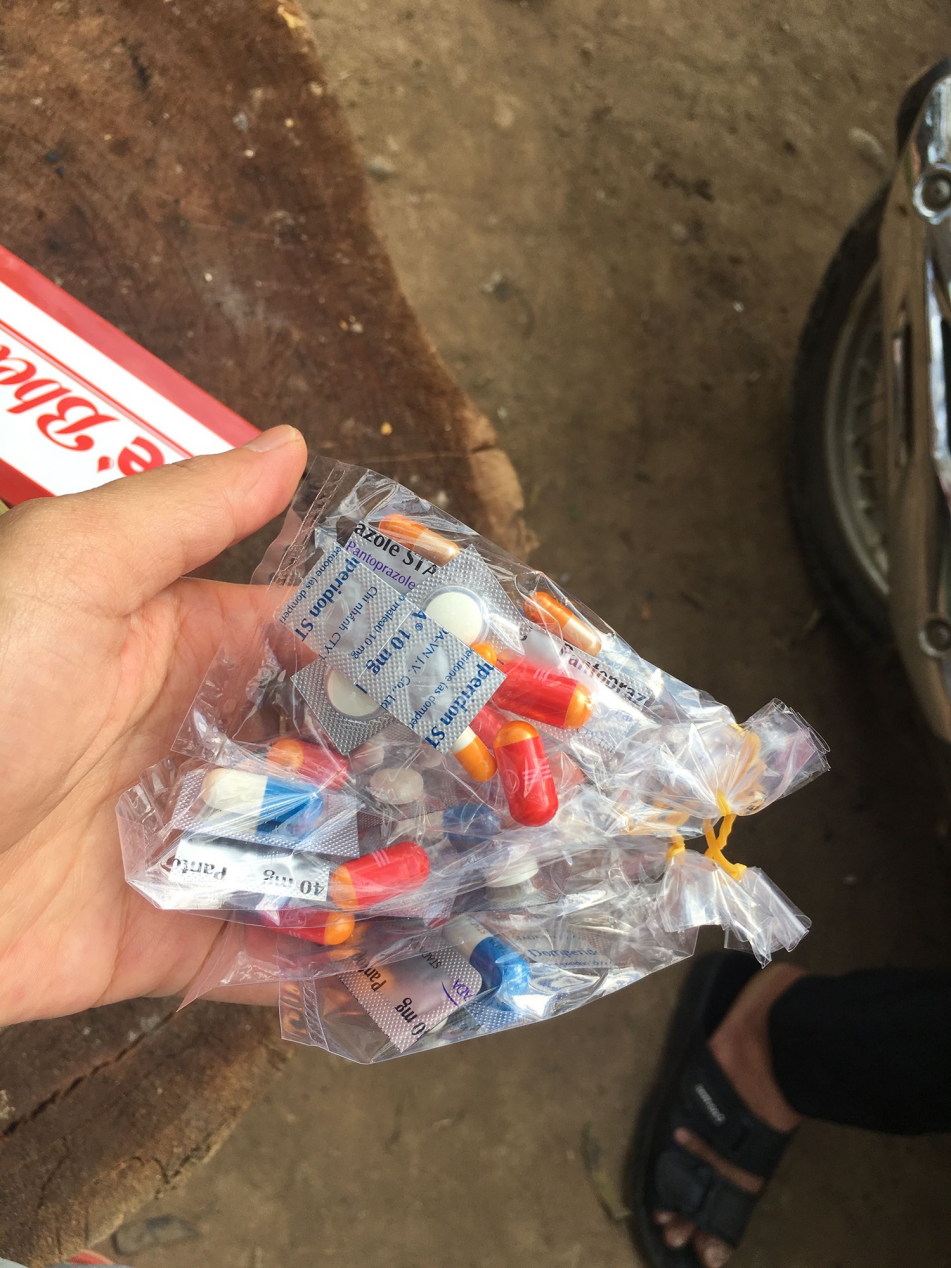
Picture of medicines bags purchased by a community member in a private pharmacy for stomach-ache and fatigue symptoms. Image credit: TTN.

Parenteral medical products, such as transfusions and injections seemed widely preferred as routes of administration by patients, as they were thought to lead to rapid symptom improvement and required more dedicated care from healthcare workers, which in turn fed into the perceived well-being of a patient. These options were also prescribed by both private and public health practitioners as they believed these administration routes could enhance the patient’s ability to recover. Nevertheless, they were more expensive and therefore the most common type of medical products consumed were oral medicine, in tablets or more seldom in instant powder. Clear distinctions were made between medication for children and for adults. Tablets were often delivered in mixed-medicine plastic bags and could contain (a combination of) labelled tablets (from cut blister packages), unlabelled tablets (in bulk), or all ground to powder, depending on the dispensers. Some dispensers (e.g. at CHCs) prepared different sachets for each type of medicine, with separate instructions per type of drug (and sachet). All these medicines were then grouped into a bigger plastic bag that was given to each patient. Other dispensers packed the mixed drugs in sachets per daily intake, while others again combined all drugs purchased with different instructions in one bag (Fig 1). Patients often had little or no idea which medicines were included in the package as it was often referred to as “medicine bag to treat fever”, “belly pain”, or “headache”, etc.

### Perceived efficacy to treat illness symptoms

The efficacy of a treatment was judged in relation to the speed with which symptoms were alleviated. Raglai informants often explained this in terms of “fast” or “strong” medicines versus “slow” or “mild” medicines. When medication was started, patients tended to take medication for one to three days in line with their capacity to pay and the other factors described hereabove. During this period, the patient evaluated the drug efficacy against the rapidity of symptom relief. If symptoms abated, treatment was considered effective and pursued (with the potential purchase of additional dosages when means allowed) until symptoms were experienced to have sufficiently decreased. The course was then considered complete and further doses were stored for future use. Treatment failure was often interpreted initially as a “bad prescription”, i.e. the wrong answer to the problem. Such a “bad prescription” could be associated with (i) poor quality of the drugs; (ii) a wrong diagnosis, i.e. the prescribed/purchased regimen was inappropriate for the disease the patient actually had; (iii) the disease being too “strong” for the prescribed medicine. The latter explanation was often linked to spiritual aetiologies, such as the spirit of the sick person being too weak or travelling too far from the physical body for the medicine to take effect.

"… *At the communal health centre, they gave me medicines, but sometimes I would still be sick after taking them for a week*. *I am not sure why, but I think the medicines they prescribed were too mild. Unfortunately, I could not afford to buy stronger medications, so I had to rely on the ones provided at the communal health centre*. *Occasionally, they would refer me to the district health centre, where they have stronger medicines and highly trained health professionals." (IDI, patient, 11/08/2018)*

### Perceived quality of medical products

Informants sometimes referred to medicines sold in grocery shops as *thuốc bậy bạ* or “nonsense medicine” implying that this kind of medicine was considered a temporary solution while waiting to see if symptoms would decline or preparing to seek care from a private or public provider further away. Informants and shop owners named these medicines “fever medicine” or “belly pain medicines”.

Among both Raglai and Kinh people, the quality of drugs from public health centres was often perceived to be lower than in private practices or pharmacies. While some people believed that the health centre medicines healed at a slower pace, others also believed these medicines could only partially heal the disease. The perception about “slow”-working domestic medicines in health centres versus “strong” and “fast”-working imported (foreign) medicines in private pharmacies or healthcare practices was shared by some staff working in health centres, pharmacies, and Raglai key informants with higher socio-economic status. These perceived benefits of foreign medicines were promoted by private providers and used as an argument to justify the higher costs.

### Factors driving diagnosis and prescribing practices of providers

#### Access to diagnostic tools

At the lowest level of care, the CHC staff reported to have limited diagnostic tools with limited staff training to use diagnostic equipment with the exception of malaria. For any signs of complication or severe illness, CHC staff (often headed by a nurse or more exceptionally a medical doctor) had to refer the patient to district or provincial levels of care, where better diagnostic technologies and trained/specialized staff were available. Private practitioners could also be found in Ninh Son who offered diagnostics capacities such as laboratory testing, medical imaging etc.

In Bac Ai district, the current public health system did not offer routine bacterial surveillance testing nor antibiotic susceptibility testing at commune- and district-level, which limited the possibility of diagnosis to clinical diagnosis and consequent prescription. Laboratory surveillance and antibiotic susceptibility testing was only possible at provincial hospital level, which was one of the least accessible/accessed levels of healthcare for Raglai due to the factors mentioned above.

*"… The communal and district health centres are required to refer suspected cases of antibiotic resistance to the provincial hospital*. *The patient must be admitted to the hospital in Phan Rang City for an antibiotic susceptibility test, which takes two days to obtain results. We rarely have patients of Raglai ethnicity, as the majority of them did not survive long enough to seek medical help from us. Raglai patients often try multiple types of antibiotics at communal health centres or private pharmacies*. *If these treatments prove ineffective, they often abandon western medicine due to its high cost." (IDI, provincial laboratory director, 18/05/2018)*

#### Access to supplies and storage

Procurement of medical products varied depending on the type of providers. Medical products (including antibiotics) at public providers were mostly manufactured locally, in Vietnam. Public structures had procedures in place for the procurement, storage, and disposal of expired drugs. Informants explained that CHC and district health centres had to submit requests for medical supplies at higher levels (from commune, to district, to provincial level) which were then procured at provincial level within the available funding following public bidding regulations. Medicine storage rooms were equipped with fans. At home practices, pharmacies or grocery shops, drugs were purchased from wholesale suppliers at district-level and storage conditions were left solely in the hand of the individual provider. Informants explained that procuring their supplies at Bac Ai district was more expensive then at Ninh Son, which was a bigger and central city, and better connected to different districts. Pharmacies could also procure supplies from large pharmaceutical companies or distributors. At grocery shops, drugs were often left in the open, with direct sunlight exposure and without measures in place to manage the expiration dates (as tablets were often stripped off or removed from their protective strips).

#### Clinical training and competence

Empirical therapy was mainly prescribed based on clinical diagnosis and seemed to commonly include prophylactic prescription of antibiotics (from observations and conversations). Trained providers from health centres, private practices, and hospitals had to compete with other prescribers, who did not necessarily have (appropriate) training in diagnosis, dispensing, storage, and use, nor imposed limitations on the types of medicines they could prescribe. Private home practitioners were often public healthcare providers with some nursing or medical training, even though the training background could vary substantially from one practitioner to another (for instance pharmacists and laboratory technicians were observed to also have home practices).

#### Perceived relevance of ABR

In public structures, regulated lists of medicines to supply and prescribe existed and seemed to be followed according to our observations. However for private/informal providers, prescription medicines were sometimes observed to be sold over-the-counter (i.e. without the customer/patient presenting a relevant prescription). Dispensers (pharmacies, grocery shops, mobile sellers) assessed which medicines to sell based on the described symptoms and known/supposed coinciding medicines as well as the financial means of the client.

Despite having heard about ABR, the interviewed healthcare workers in public structures and private pharmacies commonly shared the perception that while ABR was an issue in big cities, it was not a problem in rural areas because people living there were too poor to buy different types of antibiotics (or medicines in general) and ‘old’ first-line antibiotic treatments were still very effective in patients from rural areas. This contrasts nevertheless with the fact that ampicillin was removed from the list of medicines used by communal and district health centres a few years ago, though it was still sold in pharmacies or distributed as part of incentives for clinical studies or charity medicines donations in the study area.

## Discussion

In light of the current AMR global health threat this study explored several aspects underpinning Raglai people’s access to–and use of medical products. A theoretical framework on vulnerability underpinned this work and was further complemented by work done by Okeke, highlighting that antimicrobial use, prophylactic use, diagnostic imprecision, and interpersonal spread are key factors in the selection and dissemination of resistant strains, which can be related to poverty at the individual patient, health system, and national levels [71].

With regard to medicine use, we described different factors that impact (timely) access to care and quality medicines, as well as the logics underlying the use of medicines relating to perceived quality and type of medical products, perceived efficacy, and the explanatory logics behind treatment failure. These factors shaped the re-interpretation of the aetiology and associated re-evaluation of the therapeutic trajectory along the course of the illness [27, 30, 72–74]. In such a context, the medicine use that might be described as “irrational” from a biomedical perspective (see WHO’s definition of rational medicine use e.g. [75]) appears logical and rational from a contextualized consumer perspective [76].

The socio-cultural, economic, political, and structural context in which Raglai are embedded reveals the structural vulnerability they face. Structural vulnerability can be defined as “[a]n individual’s or a population groups’ condition of being at risk for negative health outcomes through their interface with socioeconomic, political and cultural/normative hierarchies. Patients are structurally vulnerable when their location in their society’s multiple overlapping and mutually reinforcing power hierarchies (e.g., socioeconomic, racial, cultural) and institutional and policy-level statuses […] constrain their ability to access healthcare and pursue healthy lifestyles” [77]. Our study highlights the precarious socio-economic situation of some Raglai communities, which is partially linked to their position in society as “ethnic minority” people [78, 79], leading to marginalization and sometimes even self-discrimination. This position as an “ethnic minority” affects the inter-ethnic encounter in health-seeking itineraries of ethnic minority groups in Vietnam as has been shown in other studies [80–85]. Chambers describes how the body is the most crucial asset of the poorest (referring to the body’s role in manual labour, which is often the main subsistence strategy of the poorest), yet it is more vulnerable compared to the less poor and therefore physical disabilities comprise higher personal costs [86]. Similarly, for Raglai informants the ability to do productive work was regarded as the highest priority in life, i.e. doing manual labour in the fields and everyday chores in the household.

The confluence of the above-mentioned driving factors of medical products’ use are also key for understanding the cumulative processes of (structural) vulnerability that keep the Raglai in “spirals of vulnerability” [87]. Such vulnerability can be expected to influence exposure to AMR as (i) resistant-promoting activities of some may have consequences for others; (ii) poor people are more susceptible to acquiring an infection–they have low immunity due to malnutrition and repeated and chronic infection (for example due to less access to clean water and food). Structural vulnerability will thus also have an impact on Raglai’s coping mechanisms for facing AMR: (i) people living in poverty are least able to buffer themselves from the consequences of resistance even if they may contribute less to the problems in terms of selective pressure; (ii) the burden of resistant infection is disproportionally shouldered by the less privileged who are (iii) less likely to be able to access appropriate care, ABR control interventions (as not necessarily accessible to poor people), antimicrobials, good diagnostics, good prescribers; (iv) more likely to acquire and consume partial doses, and self-medicate, as well as more likely to access substandard and counterfeit drugs originating from less regulated supply chains [71].

Raglai people’s “defencelessness” [86] to “buffer” themselves against the consequences of (resistant) infections illustrates how vulnerable populations need to be actively integrated in multisectoral interventions, policies, and research targeting AMR [76]. Raglai communities are at hightened risk of resistant infections, yet they are among the least targeted by antimicrobial use interventions, which are mostly set up in secondary and tertiary healthcare facilities and target urban populations. In addition, our findings illustrate that over-the-counter procurement and dispensing of (prescription) medicines in the private and informal sphere not only compounds to Raglai’s precarious situation but also undermines the global effort to combat suboptimal use of antimicrobials (including prescription and broader access).

### Limitations

While we looked at several social factors underlying use of medicines and potentially impacting AMR, more data on social factors related to antibiotic use in animals, prescription practices and more specific data on antibiotics is needed to gain a more holistic understanding, especially if combined with interdisciplinary data on AMR in this context (e.g., microbiological and epidemiological data). However, given the current scarcity of AMR data from this context and the time and budget limitations, this was not achievable within the scope of the current study.

## Conclusions

The precarity experienced by populations like the Raglai show the need to gather more evidence on the prevalence of AMR among vulnerable populations, as well as its underlying biological and social processes at the community level. In the meantime, access to and use of quality care and medical products could already be improved by focusing on interventions that (i) train (community) care providers and dispensers in AMR and on the risks of suboptimal (poor quality) antibiotic dispensing and use in communities and (ii) organise contextualized community sensitisation activities on these same topics; and (iii) improve active monitoring of compliance to policies regulating the procurement of medical products and their quality assurance, especially in the private sector. Nonetheless, our findings also underline the need for broader structural interventions that address the root causes of poverty and marginalisation of the Raglai people which constitute an essential dimension to tackle.

## Supporting information

S1 Checklist Inclusivity in global research. (DOCX)

S1 Table Categories of informants. (PDF)
