# Supplementary material for: Use of antimicrobials and other medical products in an ethnic minority context of South-Central Vietnam: A qualitative study of vulnerability
Source: PLOS Glob Public Health. 2024 Apr 9;4(4):e0002982. doi: 10.1371/journal.pgph.0002982 (PMC11003614; doi:10.1371/journal.pgph.0002982)
Supplement: S1 Checklist — (DOCX) [file pgph.0002982.s001.docx]

Inclusivity in global research

PLOS’ policy on inclusivity in global research aims to improve transparency in the reporting of research performed outside of researchers’ own country or community and ensures that PLOS publications reporting global research adhere to high standards for research ethics and authorship. Authors of relevant research articles may be asked to complete the questionnaire below, which outlines ethical, cultural, and scientific considerations specific to inclusivity in global research. This questionnaire may be requested when researchers have travelled to a different country to conduct research, if research uses samples collected in another country, research with Indigenous populations or their lands, or if research is on cultural artefacts. Researchers travelling to another country solely to use laboratory equipment will not normally be required to complete the questionnaire. However, the questionnaire can be requested at the journal’s discretion for any submission – if you have been requested to complete this questionnaire by the PLOS journal you submitted to, please do so.

Please complete the questionnaire below and include this as a Supporting Information file with your manuscript. Note that if your paper is accepted for publication, this checklist will be published with your article in the supporting information files. Please ensure that you reference the checklist in the main body of your manuscript. We suggest adding a subsection ‘Inclusivity in global research’ to your Methods section and adding the following sentence: “Additional information regarding the ethical, cultural, and scientific considerations specific to inclusivity in global research is included in the Supporting Information (SX Checklist)”

The questions have been designed to be applicable to a wide range of study types, and there are subsections for both human subjects research and non-human subjects research. If any of the questions are not relevant to your research please mark them as “N/A” as appropriate.

**Ethical considerations, permits and authorship**

*This section is applicable to all research types.*

Provide details as to who granted permissions and/or consent for the study to take place in the Methods section of your manuscript. This should include the names of **all** ethics boards, governmental organizations, community leaders or other bodies that provided approval for the study. If individuals provided approval refer to these people by their role or title but do not list their name(s).

Reported on page number: 10-11 (Section “Ethical approvals)

If there were any deviations from the study protocol after approval was obtained please provide details of these changes in the Methods section of your manuscript.

Reported on page number: p6 – although this doesn’t really constitute a deviation from the study protocol, since in line with the principles of iterative and flexible qualitative research design, which is the one reported in the protocol

Did this study involve local collaborators that are residents of the country where the research was conducted or members of the community studied? If you do not have any authors from said communities, please provide an explanation for this below.

Yes, the study involved local collaborators who are from the country where the research was conducted (including authors TTN, XNN, TTD).

The authors of the study do not include participants from the Raglai communities because they were the study participants who provided insights from their own setting. In this study, the initial research questions regarding the perceptions and use of antibiotics and ABR, as defined by the biomedical framework, were reframed after we commenced the fieldwork. This decision was made by the study team because for Raglai participants the concepts of antibiotics and ABR were not part of the language and required an adaptation to their reference framework.

Everyone listed as an author should meet PLOS’ criteria for authorship and all individuals who meet these criteria should be included in the author byline, rather than the acknowledgements. For further information please see the journal’s Authorship Policy.

**Human subjects research (e.g. health research, medical research, cross-cultural psychology)**

Did you obtain written informed consent from a representative of the local community or region before the research took place? How did you establish who speaks for the community? Details of written informed consent obtained from study participants should be reported separately in the Methods section of your manuscript.

Written approval was obtained from the local authorities in Ninh Thuan Province before the study started. In addition, before fieldwork, TNN sensitised local leaders in the study villages and health centres to inform them about the study and both researchers’ (TTN and MR) role in the study.

In this study, we obtained ethical approval to use oral consent for two main reasons. Firstly, the study population had a high illiteracy rate and had trust issues with written documents in Vietnamese, especially among the Raglai participants. Secondly, the study dealt with sensitive topics such as interethnic relationships, healthcare practices that are mostly carried out by health professionals from the Kinh or Cham ethnic groups and the experiences of Raglai minority patients. We ensured that all study participants were fully informed about the study, and addressed any concerns they had. We also explained their rights and provided them with an information sheet that included details about the study's objectives, data collection and use, the removal of personal identifiers from the data, and contact information in cases of complaints or decisions to withdraw their participation after an initial agreement to take part in the study

How did members of the local community provide input on the aims of the research investigation, its methodology, and its anticipated outcome(s)?

Members of the community, some of whom were also patients of local health providers, were not actively involved in defining the initial research questions, or in the dissemination plans of the research but they participated throughout the iterative process of the research. Dialogue with research communities and giving voice to people is an intrinsic part of ethnographic research. This implies that along the research process, the study design and conduct were constantly adapted based on insights from informants (including community members) along the iterative process of data collection, intermittent discussion of results and analysis, further sampling and adaptation of research questions. The fact of residing in the communities during data collection also enhanced opportunities to engage with communities at different levels than that of merely answering the research questions and allowed to gain a better understanding of the general context which was detrimental for the research.

When engaging with the local community, how did you ensure that the informed consent documents and other materials could be understood by local stakeholders?

In this study, we had the assistance of two trusted fieldworkers from the Raglai communities. Their role was to help with translation and explanation for Raglai participants, providing information about the study and how individuals could take part. Participants were provided with information based on the information sheet and both the researchers (TTN and MR) and their assistants offered additional clarification to address any concerns raised by the participants. After obtaining verbal consent from the participant, one of the researchers and, if present, a witness signed a form to confirm that they had provided information and obtained consent from the individuals participating in the study.

Will the findings of the research be made available in an understandable format to stakeholders in the community where the study was conducted (e.g. via a presentation, summary report, copies of publications, etc.)? Please provide details of how this will be achieved.

We organised briefings/information sessions for key members of the community who were identified during our fieldwork and for health staff from local health centres and NIMPE at the end of our fieldwork.

**Non-human subjects research using specimens/ animals collected as part of the study, or those housed in archival collections. Examples include archaeology, paleontology, botany and zoology.**

Did the permission you obtained from a local authority to perform the study include an agreement on access to outputs and benefit sharing? This may include procedures to enable fair distribution of the benefits and resources arising from the research performed. Please include any details of Prior Informed Consent and Benefit Sharing Agreements obtained. These may be required by field-specific regulations, for example the Convention on Biological Diversity (CBD) and the associated Nagoya Protocol.

N/A

If the material used in your study was imported, please A) provide the year it was imported and B) indicate whether permits were obtained to import/export the materials used, C) provide details of any permits obtained. If this information is not available, please indicate this.

N/A

If you used archival specimens, please state how the material used in your study was acquired by the institute it is held in and provide details of any permits obtained for the original excavations/ sample collection. If this information is not available, please indicate this.

N/A

How was the potential cultural significance of the materials collected in your study to local communities considered in your research design? Were Indigenous peoples and/or local researchers and institutions involved with archaeological excavations / collection of specimens? If so, please provide a description of their involvement.

N/A

If your manuscript includes photographs of human remains please indicate whether authors obtained permission from descendants or affiliated cultural communities to do so.

N/A
