# Supplementary material for: Use of antimicrobials and other medical products in an ethnic minority context of South-Central Vietnam: A qualitative study of vulnerability
Source: PLOS Glob Public Health. 2024 Apr 9;4(4):e0002982. doi: 10.1371/journal.pgph.0002982 (PMC11003614; doi:10.1371/journal.pgph.0002982)
Supplement: S1 Table — (PDF) [file pgph.0002982.s002.pdf]

Supplementary Table 1. Categories of informants.

Categories of informants		
Health care providers / dispensers	Public sector	Medical doctors, nurses, midwifes, pharmacists, pharmacist assistants, laboratory specialists, laboratory technicians, village health workers
	Private sector	Pharmacists, pharmacy assistants, home practitioners, grocery shop owners
	Traditional sector	Shamans
Animal health care providers / dispensers	Public sector	Veterinarian officers, veterinarian assistants
	Private sector	Sales managers, veterinarian customer service, experienced farmers
Community leaders	Government	Government officials, forest protection guards, chiefs of villages
	Traditional leaders	Shamans, respected "wise" & knowledgeable individuals by community members
Community members	Farmers, plantation workers, veterans, current and former (parents of) patients, female and male youth, female and male adults, female and male elderly "tree workers"/loggers, shop keepers, petty/'snacks' sellers, restaurant owners, mobile food sellers	
